# Perioperative Exercise Testing in Pregnant and Non-Pregnant Women of Reproductive Age: A Systematic Review

**DOI:** 10.3390/jcm13020416

**Published:** 2024-01-11

**Authors:** Madeleine G. Spicer, Alicia T. Dennis

**Affiliations:** 1 Department of Obstetrics and Gynaecology, Alice Springs Hospital, Alice Springs, NT 0870, Australia; 2 Department of Anaesthesia, Pain and Perioperative Medicine, Joan Kirner Women’s and Children’s Hospital, Western Health, St Albans, VIC 3021, Australia; adennis@unimelb.edu.au; 3 School of Medicine, Faculty of Health, Deakin University, Melbourne, VIC 3125, Australia; 4 Departments of Critical Care, Obstetrics and Gynaecology, University of Melbourne, Melbourne, VIC 3010, Australia; 5 Brigham and Women’s Hospital, Harvard Medical School, Boston, MA 02115, USA

**Keywords:** functional capacity, functional exercise testing, perioperative assessment, healthy females, physiology

## Abstract

Background: Women have classically been excluded from the development of normal data and reference ranges, with pregnant women experiencing further neglect. The incidence of Caesarean section in pregnant women, and of general operative management in young women (both pregnant and non-pregnant), necessitates the formal development of healthy baseline data in these cohorts to optimise their perioperative management. This systematic review assesses the representation of young women in existing reference ranges for several functional exercise tests in common use to facilitate functional assessment in this cohort. Methods: Existing reference range data for the exercise tests the Six Minute Walk Test (6MWT), the Incremental Shuttle Walk Test (ISWT) and Cardiopulmonary Exercise Testing (CPET) in young women of reproductive age were assessed using the MEDLINE (Ovid) database, last searched December 2023. Results were comparatively tabulated but not statistically analysed given underlying variances in data. Results: The role of exercise testing in the perioperative period as an assessment tool, as well as its safety during pregnancy, was evaluated using 65 studies which met inclusion criteria. Conclusion: There is a significant lack of baseline data regarding these tests in this population, especially amongst the pregnant cohort, which limits the application of exercise testing clinically.

## 1. Introduction

Exercise testing is a useful method for cardiorespiratory functional assessment. Preoperatively, it assists in risk-stratifying patients into operative and non-operative management groups and can improve surgical outcomes [1]. As functional walking tests are straightforward and require little equipment, they are commonly employed as a morbidity assessment tool in patients with cardiac failure, respiratory disease, or other chronic conditions [2]. As such, the reference values for older patients with various chronic diseases are well characterised; however, the establishment of baseline data in young, healthy women has been relatively overlooked, even with demonstrable need and reassuring data regarding the safety of exercise during pregnancy.

Young women also become acutely and chronically unwell and often require surgery; thus, the use of simple and rapid functional exercise tests could greatly benefit this population. Exercise assessment of chronic lung disease (such as cystic fibrosis) and congenital heart disease in a paediatric population has been shown to be useful [3]. As more children with congenital conditions live into adulthood and become pregnant, reference ranges for young women could maintain their follow up. These data could also guide the management of perioperative illnesses of pregnancy (such as preeclampsia and heart failure) and facilitate preoperative optimisation (pre-pregnancy and antenatally) in anticipation of potential Caesarean section surgery—a concept that is rarely considered, despite the operation’s prevalence [4]. According to the World Health Organisation (WHO), 21.1% of women globally underwent Caesarean section between the years of 2010–2018, and this figure is projected to rise to 28.5% by 2030 [5]. Of an anticipated 38 million annual Caesarean sections in 2030, 33.5 million of these will be performed in low- and middle-income countries [5]. In these countries, pregnant women are more likely to have complex antenatal issues such as infectious diseases, congenital conditions and lesser access to regular antenatal care. Implementing routine exercise assessment could greatly reduce morbidity and mortality in this context.

Furthermore, regular exercise throughout pregnancy is known to improve perinatal outcomes for mother and child. A meta-analysis regarding the effects of maternal exercise on perinatal and childhood growth found that in normal-weight mothers undergoing exercise intervention, the rate of preterm birth (PTB) decreased by 15%, and the rates of small-for-gestational-age (SGA) and large-for-gestational-age (LGA) babies both decreased by 17%. In mothers who were overweight or obese, even more significant impacts were seen, with rates of PTB reduced by 33%, SGA by 27% and LGA by 55% [6]. Direct reductions could be extrapolated from this in rates of neonatal morbidity and mortality from prematurity, severe perineal trauma from the birth of LGA babies, elective Caesarean delivery for SGA babies, neonatal intensive care costs and bed occupancy and maternal psychological birth trauma from complicated birth. This same meta-analysis also demonstrated a reduction in risk of childhood obesity for the children of normal-weight exercising mothers, which improves longer-term health for the next generation [6].

Antenatal exercise also has known benefits in reducing the risk of excessive gestational weight gain, and the prevention of gestational diabetes and hypertensive disorders of pregnancy. The prevention of these pregnancy-associated maternal conditions is essential to modify the far-reaching subsequent risk of chronic disease for women as they age [7].

Outside of pregnancy, emerging data which demonstrate the influence of exercise on neuroplasticity further supports the recommendation for adequate physical activity for adults. In a study of fifteen untrained individuals who underwent a twelve-week aerobic training program, Moscatelli et al. discovered that not only was there a significant increase in maximal oxygen consumption (VO_2max_), there was also a decrease in resting motor threshold (*p* < 0.001) and motor-evoked potential latency (*p* < 0.001) and an increase in motor-evoked potential amplitude (*p* < 0.001), reflecting changes in cortical excitability [8]. The known effects of exercise on mood also support the establishment of an antenatal exercise prescription which carries through to peripartum and postpartum period, as this is protective against the symptoms of postnatal depression and reduces fatigue levels for new mothers [9].

This review examines the application of the Six Minute Walk Test (6MWT), the Incremental Shuttle Walk Test (ISWT) and Cardiopulmonary Exercise Testing (CPET) in the young female population in order to examine the existing data to inform exercise assessment of this cohort. Incremental exercise testing (such as CPET) is the gold-standard for assessing maximal aerobic power through measurement of VO_2max_ [10]. It has therefore been selected for appraisal to inform baseline data for maximal capacity. The 6MWT and ISWT were also selected as they are considered to be the best-validated of the field tests (correlating well with VO_2max_ when compared to incremental exercise testing) [11,12]. Furthermore, they are easily implemented. Other timed walk tests (such as the Twelve or Two Minute Walk Test) were not explored, as the 6MWT is generally regarded as “a sensible compromise” [13]. The high correlation coefficients of the three timed tests indicate that they measure exercise tolerance similarly, and the Six Minute approach encompasses the reproducibility of the shorter test with the improved discrimination of the longer test [13]. Furthermore, as the Endurance Shuttle Walk Test (ESWT) is a second-line test derived from the ISWT, it was not explored as a primary test for the proposed clinical application.

The 6MWT, whilst a well-validated predictor of functional status, is a self-paced exercise test and is thus less physically rigorous than intensity-matched externally paced exercise due to behavioural influences over exertion which reduce energy expenditure [2,14]. This may affect the repeatability of data due to a lack of standardisation across participants, and even within a single participant’s trials. As such, an externally paced test, whereby a marker dictates walking speed, may standardise effort and better assess maximal physical capacity as there is less ability to exert behavioural responses to disrupted homeostasis [14,15]. However, no significant difference in the validity of exercise testing has been found by using self-paced versus externally paced exercise. Self-paced exercise testing does emulate the context of Activities of Daily Living (ADLs) well [16]. However, from a perioperative perspective, the use of an externally paced functional exercise test such as the ISWT or CPET could provide accurate information about true maximal capacity.

Through assessment of the data available regarding CPET, the 6MWT and the ISWT, and subsequent identification of areas lacking investigation, the application of maximal and submaximal exercise testing in this population can be utilised clinically to inform practice, monitor patient progress and optimise in a medical and perioperative setting.

## 2. Materials and Methods

As a review of existing published literature, no institutional ethics approval was required. No systematic review registration was sought.

**Figure 1:**
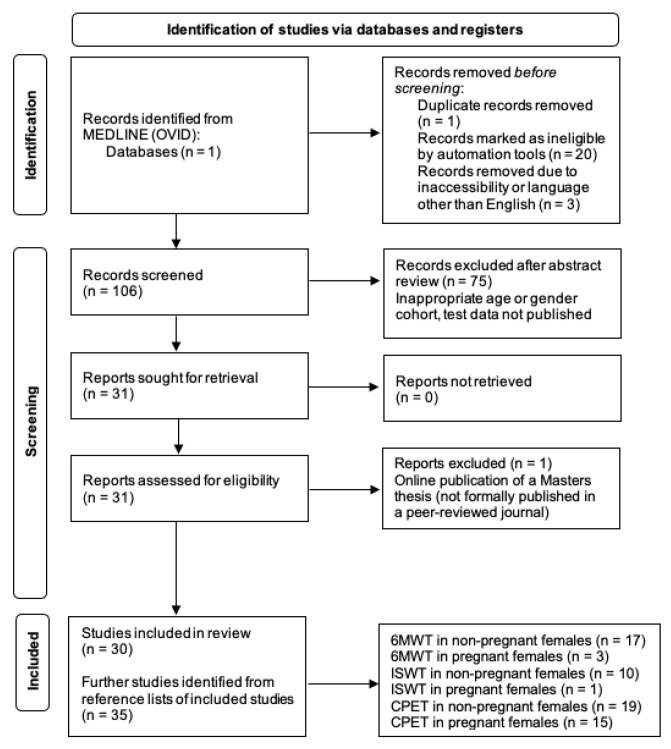
Search strategy to identify studies for inclusion regarding the Six Minute Walk Test (6MWT), Incremental Shuttle Walk Test (ISWT) and Cardiopulmonary Exercise Testing (CPET) within the domains of female sex and reproductive age. *n*: number.

The search strategy followed the PRISMA guidelines (Figure 1) [17]. One reviewer undertook an initial review in April 2019, with supplementary reviews in November 2021 and December 2023. The MEDLINE (Ovid) database was searched using the same strategy three times to assess each exercise test. Keywords included the name of the test (either “Six Minute Walk Test”, “Incremental Shuttle Walk Test” or “Cardiopulmonary Exercise Testing”) and “reference range”, with limits encompassing “adult 19–44 years”. Due to limited returned results, further references were gleaned from the reference lists of identified articles. All types of studies that reported the basic parameters of the test in question and general cohort characteristics were included. These records were hand-searched to ensure they met language criteria (English) and included female participants (either exclusively or in a combined cohort with males).

The studies reporting raw data are presented as comparative tables. Any studies with mean age of female participants aged >45, or studies with mean patient body mass index (BMI) ≥ 30, were excluded from tables. No analytical methods were used to combine these studies as underlying variances in data grouping and presentation precluded this.

The subsequent composition of this systematic review was guided by the PRISMA checklist.

## 3. Results

### 3.1. The Six Minute Walk Test (6MWT) in Females of Reproductive Age

#### 3.1.1. Non-Pregnant

Fifteen studies were identified which presented 6MWT reference ranges in the target population (Appendix A Table A1), and a further three studies were used for reference (but had mean age above the target population). Publications ranged from 1998 to 2021.

As 6MWT reference ranges in healthy women of reproductive age are less reported, it is also unclear what the relevant Minimal Clinically Important Difference (MCID) is for this cohort; however, consensus on the MCID in older, chronically ill patients is approximately 30 metres (m), and that a decrease in 6MWD of ≥30 m signifies increased mortality in the next twelve months [18,19]. However, reference ranges developed from older patients are not suitable predictors for younger cohorts as both the prevalence of medical comorbidities and the reduction in skeletal muscle mass and strength in the aged population reduce the average 6MWD [20]. Studies have demonstrated a strong correlation between increasing age and decreasing walk distance (*p* < 0.005 in five studies) [21,22,23,24,25]; this effect, however, is less pronounced when comparing subsets of younger adults by age (with no significant difference in 6MWD when comparing an 18–50 year old cohort) [26]. Oliveira et al. postulate that 6MWD decreases by approximately two metres with each year of increasing age, and Dourado et al. found a decline of 9.3% per decade in women (and 9.5% per decade in men) [27,28].

Measures including weight, BMI, height and biological sex also significantly affect 6MWT outcomes [21,29,30,31,32]. The exclusion of overweight and obese women from reference range development is supported by research which shows that obese women walk a shorter distance during any given exercise, due to increased workload from excess weight [20]. Increased BMI correlates with decreased 6MWD, with *p* = 0.02 (r = 0.145) to *p* < 0.05 (correlation coefficient of −0.24); increased weight has a similar effect, with *p* < 0.001 [21,33]. Male sex and increased height are associated with farther distances walked (*p* < 0.01) [21,23,24,34]. Female sex is associated with changes to other test parameters, including a higher mean resting, walking and maximum heart rate [22]. Higher levels of reported physical activity also predict farther walking distance (*p* < 0.01 and r = 0.25; *p* < 0.0001) [33,35,36].

#### 3.1.2. Pregnant

Pregnancy itself can impact 6MWT outcomes (as demonstrated in three studies across 2010 to 2018; Table 1). Comparative data confirm that 6MWD decreases with increasing gestation; a cohort of 100 women at 21 weeks’ gestation (SD 1.8 weeks) walked a mean distance of 548 ± 80.9 m, compared to 300 women with mean gestation of 37 weeks (SD 1.3 weeks) who walked a mean distance of 488 ± 94.9 m—an average decrease of 60 m from early to late pregnancy [37,38]. A study performed in thirty-seven pregnant women with mean gestational age 22 ± 1 weeks demonstrated that the predicted VO_2max_ from the 6MWT was moderately correlated to the measured VO_2max_ as assessed by graded maximal treadmill test (r = 0.40, *p* = 0.016) and strongly correlated in the control group of ten non-pregnant women (r = 0.80, *p* = 0.006). The authors concluded that the 6MWT could be utilised to assess fitness in mid-trimester pregnancy but is not a precise measure [39]. One study made limited comment about perinatal outcomes, stating that 8 of 200 women (4%) developed preeclampsia [38].

Preeclampsia during pregnancy has an added effect on the test. A group of 74 primiparous women with gestational age ≥ 24 weeks was assembled, with half of the women having preeclampsia and half experiencing a normal pregnancy. The women with preeclampsia walked significantly less far (*p* = 0.001) [40]. This may be attributable to higher gestational BMI (28 ± 5 kg/m^2^ in healthy pregnant women versus 31 ± 4 kg/m^2^ in women with preeclampsia; *p* = 0.01) and cardiovascular changes and airway oedema, which may decrease functional capacity [40]. Furthermore, women with preeclampsia classified as having “inadequate BMI” (defined as overweight or obese) walked significantly less far than women with preeclampsia with “adequate BMI” (defined as normal); this outcome was not seen in healthy women of normal versus high BMI. Exercise may modify the risk of developing hypertensive diseases of pregnancy; in one study, upkeeping regular leisure time physical activity (LTPA) before 20 weeks’ gestation significantly reduced the risk of preeclampsia (adjusted RR 0.67, 95% CI (0.46–0.96)) and gestational hypertension (aRR 0.75, 95% CI (0.54–1.05)). The relative risk of developing either condition decreased with increasing LTPA (aRR for low, moderate and high energy expenditure in preeclampsia: 1.00, 0.77 and 0.57, *p* = 0.01; and for gestational hypertension: 1.00, 0.80 and 0.71, *p* = 0.08) [41]. Recurrent hypertensive diseases of pregnancy have severe long-term impacts on cardiovascular disease in women, with an adjusted hazard ratio of 3.30 for future ischaemic heart disease (95% CI (2.02–5.40)) and 5.10 for stroke (95% CI (2.62–9.92)) [42].

Additionally, parity may influence 6MWT results: a study of North African women aged 45–59 years subdivided into two groups—low parity (≤5 births) or high parity (≥6 births)—demonstrated a link between increased parity and decreased 6MWD (589 ± 60 m, parity ≤ 5; 555 ± 57 m, parity ≥ 6; *p* < 0.05) [43]. This is postulated to be due to repeated oxidative stress and hormonal changes causing aerobic incapacity. In contrast, the Bogalusa Heart Study, which assessed 761 women about their reproductive history and physical function in later life, found that women with high parity had better functioning than nulliparous women [44]. Women were primarily assessed with the Short Physical Performance Battery (SPPB), but the 6MWT was also performed. In this cohort (mean age 47.7 years; 58% post-menopausal), nulliparity was associated with decreased walking distance compared to parous women (mean 6MWD 414 ± 80 m; differences not stated). Here, nulliparity was taken to represent “infertility”, and the authors posit that “infertility may indicate overall worse health”.

**Table 1:** Reference range studies for the Six Minute Walk Test in healthy pregnant women.

Study	Female Participants (*n*)	Age (Years)	Gestation (Weeks)	6MWD (m)	Resting Baseline Variables
Dennis 2018 [37]	100	31 ± 5.2	21 ± 1.8	548 ± 80.9	SBP (mmHg) 114 ± 10.1 DBP (mmHg) 71 ± 8.8 HR (bpm) 81 ± 11.1 SpO_2_ (%) 98 ± 0.8 RR (brpm) 16 ± 2.1
Dennis 2018 [38]	300	31 ± 4.2	37 ± 1.3	488 ± 94.9	SBP (mmHg) 112 ± 10.2 DBP (mmHg) 72 ± 8.6 HR (bpm) 85 ± 10.8 SpO_2_ (%) 98 ± 0.9 RR (brpm) 18 ± 5.7
da Silva 2010 [40]	37	22 (18, 24)	37 (33, 38)	497 ± 38	SBP (mmHg) 110 (100, 110) DBP (mmHg) 70 (60, 70) HR (bpm) 84 ± 15 SpO_2_ (%) 98 (97, 98)

*n*: number of female participants; 6MWD: Six Minute Walk Distance; m: metres; SBP: systolic blood pressure; DBP: diastolic blood pressure; HR: heart rate; SpO_2_: peripheral oxygen saturation; RR: respiratory rate; mmHg: millimetres of Mercury; bpm: beats per minute; brpm: breaths per minute. Data are mean ± SD, or median (IQR).

### 3.2. The Incremental Shuttle Walk Test (ISWT) in Females of Reproductive Age

#### 3.2.1. Non-Pregnant

Ten studies (from 2011 to 2020) were identified which explored the ISWT in the target population (eight of these are tabulated (see Appendix A Table A2), with two studies excluded from the table due to mean age above the target population). MCID is not well-defined, but studies performed in chronically ill cohorts (including bronchiectasis, COPD and cardiac disease patients) suggest a loss of approximately 35 to 70 m is significant [45,46,47].

Like the 6MWT, candidate sex has a significant impact on ISWT performance, as exemplified by Agarwal et al. [48]. This study provided results for age-grouped young non-pregnant Indian women, demonstrating that walking distance for female participants peaked between 20 and 30 years. The 17–20 year age group walked a mean (SD) 694.78 (61.14) m; 21–30 year olds walked 730.00 (48.67) m; 31–40 year olds walked 664.10 (44.11) m; and 41–50 year olds walked 624.32 (41.13) m. Comparatively, walking distance for male participants peaked at 30–40 years. After age 40, differences between the sexes began to decrease, attributable to an age-related reduction in muscle mass and strength and decreased maximal oxygen uptake occurring [49]. Even when comparing postmenopausal women (defined as >48 years) to premenopausal women, a decrease in Incremental Shuttle Walk Distance (ISWD) of 29% can be seen [48]. The influence of age on ISWD consistently demonstrates a *p*-value of <0.001 [48,50,51,52], with Agarwal demonstrating a correlation with age of r = 0.73 and *p* = 0.000 [48].

Unfortunately, the majority of ISWT data are grouped by age only, focusing on this as the main variable influencing the ISWD (Appendix A Table A2) [48,50,51,52,53,54]. Subsequently, these published reference values, which combine both male and female data to report a mean value according to age group, are inherently less accurate as they will overestimate female performance. Height and male sex significantly increase the distance walked (*p* < 0.001) [48,50,51,52]. Factors such as greater absolute muscle strength in males, as well as increased height and leg length (*p*-value < 0.05), make biological sex a significant variable to take into account [49,51]. Furthermore, one study found that male sex was associated with reaching maximal effort (defined as HRmax > 90% of age-predicted HRmax), whilst female sex was not [55]. Only one study found no difference in exercise capacity between the sexes when correction for age was performed [51].

Forced expiratory volume in one second (FEV1), resting blood oxygen saturation levels (SpO_2_), quadriceps muscle voluntary contraction (QMVC), total energy expenditure, Duke Activity Status Index (DASI) (all *p* < 0.001) and leg length, resting heart rate (HR) and total steps (all *p* < 0.05) all affected distance walked according to Harrison et al. [51]. However, this study did not indicate how changes in these variables affected distance walked, and only reported that these factors begot significant differences; furthermore, these data were not disaggregated into female and male subsets.

Ethnic background may also impact distance walked; Orme et al. found that the ISWD of South Asian adults was significantly less than that of Caucasian British people (451 ± 143 vs. 575 ± 180 m, *p* < 0.001) [56]. Multiple factors including height and leg length, as dictated by genetic heritage, likely contribute to this result; several studies have attributed increased height to increased ISWD (*p* < 0.001) [48,50,51,52].

#### 3.2.2. Pregnant

No available literature appraising healthy pregnant women in the ISWT could be identified. However, one study (from 2010) assessed ISWT performance in pregnant women with pulmonary hypertension. Kiely et al. performed the ISWT in a small cohort of nine women with known pulmonary hypertension at “baseline”, ostensibly at their earliest point of presentation during their pregnancy, and compared later intrapartum ISWD results (Appendix A Table A3) [57]. No clear trends can be ascertained due to a small dataset, incomplete measurements for every participant and the confounding factor of medication introduction (the main aim of the study being to assess if improved medication compliance would improve exercise tolerance). The authors state that no maternal mortality occurred, but one woman died at four weeks postpartum due to self-ceasing therapy for pulmonary hypertension. All children were living at median 3.2 years of follow-up.

### 3.3. Cardiopulmonary Exercise Testing (CPET) in Females of Reproductive Age

#### 3.3.1. Non-Pregnant

CPET provides a comprehensive picture of exercise tolerance as an integrative assessment of many body systems. It analyses expired gases during exercise, pinpointing the VO_2max_, a measure of the maximal oxidative metabolism which can be achieved by a patient’s large muscle groups. This is subsequently the gold-standard value for evaluating cardiorespiratory fitness [10]. However, exercise is often limited by symptoms or exhaustion before the VO_2max_ is reached; thus, VO_2peak_ is often used as an estimate of this and is an equally useful surrogate value. Another measurable outcome is the anaerobic threshold (AT), an estimation of the onset of metabolic acidosis caused, in part, by increasing arterial blood lactate concentrations during exercise [10]. This represents the maximum intensity at which exercise can be performed using an entirely aerobic energy source and is a good indicator of fitness [10]. As such, many CPET studies focus on determining reference values for the important parameters of VO_2max_ (or VO_2peak_) and AT.

Eighteen studies from 1994 to 2021 were included regarding normal CPET outcomes for females of reproductive age. Data from sixteen of these studies have been tabulated (see Table 2 and Table 3), and the remaining two studies did not publish VO_2max_ and AT data comparably (and have thus been cited separately in the text). The MCID for peak work rate in ramp-type protocols has been cited as 4 watts (W) in COPD patients, and the MCID for AT as 2 mL O_2_/kg/min in abdominal aortic aneurysm patients [58,59]. As analysis of the SHIP study found, it is important to include BMI (as well as sex and age data) to predict VO_2peak_ more accurately through CPET [60]. Additionally, sex-specific VO_2max_ prediction equations should be utilised, as VO_2max_ is significantly higher in males than in females (29.4 ± 10.5 vs. 24.2 ± 9.2 mL/kg/min; *p* < 0.01) [61].

Table 2 demonstrates the profound effect of regular physical activity on the CPET profile, as investigated by Herdy et al. [62]. While VO_2max_ trends downwards consistently across increasing age groups for both active and sedentary women (with “active” defined as partaking in over 30 min of physical activity three times weekly or more), active older females still maintain a VO_2max_ considerably above that of younger sedentary women (45.4 ± 6.8 mL/kg/min in active 35–44 year olds, as opposed to 35.6 ± 5.7 mL/kg/min in sedentary 15–24 year olds). The AT reflects the same trend. In the long term, even moderate levels of activity are protective against a deterioration in VO_2max_ [63]. Comparison of VO_2max_ by age group in young women is shown in Table 3 [63,64,65,66,67,68,69,70,71,72,73,74,75,76,77,78,79], demonstrating a trend towards a reduction in VO_2max_ with increasing age.

**Table 2:** Cardiopulmonary Exercise Testing-measured values in active and sedentary non-pregnant females.

	Outcome Measured	Group 1 (Ages 15–24)	Group 2 (Ages 25–34)	Group 3 (Ages 35–44)
Active females	Number of women	343	597	427
	VO_2max_ (mL/kg/min)	50.6 ± 7.3	47.4 ± 7.4	45.4 ± 6.8
	AT (mL/kg/min)	33.3 ± 7.4	30.9 ± 6.8	30.1 ± 6.4
	O_2_ pulse (mL/beat)	19.6 ± 3.7	20.0 ± 3.6	19.9 ± 3.4
	VE (L/min)	115.6 ± 25.0	115.6 ± 25.6	113.3 ± 23.2
	RER	1.22 ± 0.4	1.20 ± 0.2	1.19 ± 0.3
	HR_max_ (bpm)	194 ± 9	184 ± 10	178 ± 9
Sedentary females	Number of women	85	188	157
	VO_2max_ (mL/kg/min)	35.6 ± 5.7	34.0 ± 4.8	30.0 ± 5.4
	AT (mL/kg/min)	21.5 ± 5.2	21.3 ± 4.4	19.1 ± 4.3
	O_2_ pulse (mL/beat)	10.9 ± 2.1	10.7 ± 1.8	10.2 ± 2.0
	VE (L/min)	70.7 ± 17.6	69.9 ± 15.7	64.8 ± 15.0
	RER	1.22 ± 0.3	1.22 ± 0.2	1.20 ± 0.3
	HR_max_ (bpm)	194 ± 8	185 ± 10	179 ± 12

VO_2max_: maximal oxygen consumption; AT: anaerobic threshold; O_2_ pulse: oxygen pulse; VE: ventilation per minute; RER: respiratory exchange ratio; HR_max_: maximum heart rate. mL: millilitres; L: litre; kg: kilogram; min: minute; bpm: beats per minute. Data are mean ± SD. Data adapted from Herdy (2011) [62].

**Table 3:** Maximal oxygen consumption values of non-pregnant females performing Cardiopulmonary Exercise Testing.

Study		Age (Years)		
		20–29	30–39	40–49
Liu 2021 [77]	*n*	37 ^+^	-	-
	VO_2peak_ (mL/kg/min)	30.4 ± 5.7	-	-
Almakhaita 2019 [74]	*n*	102 ^#^	0	0
	VO_2max_ (mL/kg/min)	27.4 ± 4.1	-	-
Zubac 2021 [63]	*n*	14 ^%^	14 ^}^	-
	VO_2max_ (mL/kg/min)	40.0 ± 1.3	37.2 ± 2.1	-
Bar-Yoseph 2019 [76]	*n*	11 ^&^	-	-
	VO_2max_ (mL/kg/min)	43.4 *±* 10.0	-	-
Rossi Neto 2019 [70]	*n*	732	2028	1985
	VO_2max_ (mL/kg/min)	36.9 ± 6.6	36.0 ± 7.0	34.7 ± 7.1
Triantafyllidi 2019 [72]	*n*	4	12	21
	VO_2max_ (mL/kg/min)	30 ± 5	24 ± 3	23 ± 3
Van de Poppe 2019 [78]	*n*	-	595	-
	VO_2peak_ (mL/kg/min)	-	33.8 ± 6.8	-
Fernandez 2018 [73]	*n*	15	14	14
	VO_2peak_ (L/min)	4.6 ± 2.4 *	4.6 ± 2.4 *	4.6 ± 2.4 *
Kaminsky 2015 [64]	*n*	410	608	843
	VO_2max_ (mL/kg/min)	37.6 ± 10.2	30.9 ± 8.0	27.9 ± 7.7
Buys 2015 [79]	*n*	-	534 ^$^	-
	VO_2peak_ (mL/kg/min)	-	31.5 ± 7.1	-
Almeida 2014 [75]	*n*	-	-	1209 ^~^
	VO_2peak_ (L/min)	-	-	2.16 ± 0.82 ^=^
Loe 2014 [71]	*n*	92	203	249
	VO_2peak_ (mL/kg/min)	42.8 ± 7.6	39.6 ± 7.0	37.8 ± 7.0
Edvardsen 2013 [65]	*n*	37	63	87
	VO_2max_ (mL/kg/min)	40.3 ± 7.1	37.6 ± 7.5	33.0 ± 6.4
Aspenes 2011 [66]	*n*	247	411	610
	VO_2max_ (mL/kg/min)	42.9 ± 7.6	39.8 ± 6.8	37.9 ± 7.0
Neder 2001 [67]	*n*	20	20	20
	VO_2max_ (L/min)	1.7 ± 0.2 ^^^	1.7 ± 0.2 ^^^	1.3 ± 0.14
Davis 1997 [68]	*n*	20	21	20
	VO_2max_ (L/min)	1.9 ± 0.3	1.8 ± 0.3	1.6 ± 0.2
Fairbarn 1994 [69]	*n*		60 ^>^	
	VO_2max_ (mL/kg/min)	43.9 ± 9.6	43.9 ± 5.4	35.9 ± 7.3

*n*: number of female participants; VO_2max_/VO_2peak_: maximal oxygen consumption. mL/kg/min: millilitres per kilogram per minute; L/min: litres per minute. Data are mean ± SD. ^+^: Data collected from women with mean age 20.8 ± 0.7 years. ^#^: Age group 19–25, with mean age 19.9 years. ^%^: Age group 19–30, with mean age 25 ± 1 years. ^}^: Age group 36–53, with mean age 45 ± 2 years. ^&^: Age group with mean 26.3 ± 4.6 years. *: Pooled age group with mean age 39.0 ± 11.8 years. ^$^: Mean age 35.1 ± 9.78. ^~^: Mean age 41.3 ± 14.5 years. ^=^: Pooled male and female data. ^^^: Data reported as a 20–39 year age group. ^>^: 60 women in the 20–49 year age group.

Other important cardiovascular variables can be measured with CPET, such as HR and BP. Average HR and systolic blood pressure (SBP) in females aged 20–29 were assessed by CPET at rest, at the AT and at peak exercise (Appendix A Table A4). In this study by Itoh et al., women who exercised more than twice weekly were excluded, and BMI was limited to 17.6–28.6 kg/m^2^, so the resultant values are quite representative of average healthy women who do not formally train [80].

#### 3.3.2. Pregnant

Thirteen publications, from 1988 to 2021, were identified which profiled CPET results in pregnant women; eight of these demonstrate VO_2max_ changes at different gestations (Table 4). The remaining data are explored in the text and in Appendix A, Table A5. Of these publications, perinatal outcomes were not explored but two made reference to subsequent normal pregnancy outcomes [81,82].

As Wowdzia and Davenport illustrate, pregnancy-induced physiological adaptations affect the interpretation of measured CPET variables [83]. They emphasise the importance of establishing standard criteria for pregnant populations for CPET testing and suggest accepting a higher resting HR cut-off (up to 120 bpm) and BP criteria (SBP ≤ 140 mmHg and DBP of ≤90 mmHg) and formulating a pre-exercise carbohydrate intake guideline (as pregnancy changes hepatic glycogen storage capacities) [84]. As Bijl et al. demonstrate, altered responses to exercise during pregnancy are present as early as the first trimester when compared to non-pregnant controls (including increased ventilation-perfusion mismatch and increased dead space ventilation) [85]. Furthermore, Boardman et al. identified a significant change in resting left ventricular end-diastolic volume (LVEDV) (77.3 ± 15.1 vs. 83.8 ± 17.2 mL, *p* = 0.008) and respiratory exchange ratio (RER) (1.05 ± 0.05 vs. 1.11 ± 0.05, *p* = 0.01) between 14 and 24 weeks’ gestation [86]. The authors attribute the significance of the RER potentially to test familiarity, but altered LVEDV may reflect known changes to cardiac function during pregnancy (with one study citing an average 23% increase in LVEDV during pregnancy (*p* < 0.001)) [87]. These data further reinforce the importance of developing pregnancy-appropriate CPET data, including trimester-specific reference ranges.

A systematic review by Melzer et al. assessed the several existing studies which directly measured maternal VO_2max_ rather than extrapolating using maximal heart rate-VO_2max_ curves and discovered that there is no significant difference in VO_2max_ between pregnant and non-pregnant women during maximal exercise [88]. Some studies which compared pre-pregnancy exercise values to those several weeks postpartum detected a detraining effect associated with pregnancy; it is unknown whether this effect is due to decreased exercise during pregnancy in these women or due to the state of pregnancy itself [89]. South-Paul assessed 11 women with mean age of 27.6 ± 2.2 years, measuring their VO_2max_ prior to pregnancy and 4–8 weeks postpartum. A pre-pregnancy VO_2max_ of 32.5 ± 1.7 mL/kg/min and a postpartum VO_2max_ of 30.5 ± 2.0 mL/kg/min were found [89]. These values are consistent with (but slightly lower than) average CPET studies in non-pregnant females.

**Table 4:** Reference range studies for Cardiopulmonary Exercise Testing in healthy pregnant women.

Study	Participants (*n*)	Age (Years)	Average Gestation (Weeks)	VO_2max_ (L/min)
Serial studies				
Boardman 2015 [86]	10	35 ± 4	14 weeks’ gestation	2.85 ± 0.63 *
			24 weeks’ gestation	2.66 ± 0.29 *
Treuth 2005 [90]	63	31 ± 4	Before pregnancy	2.08 ± 0.44
			6 weeks postpartum	1.73 ± 0.35
			27 weeks postpartum	1.84 ± 0.40
Spinnewijn 1996 [91]	11	33 ± 1	32 weeks’ gestation	2.36 ± 0.12
			10 weeks postpartum	2.29 ± 0.10
McMurray 1991 [92]	10	Not reported	25 weeks’ gestation	1.64 ± 0.12
			35 weeks’ gestation	1.48 ± 0.11
			10 weeks postpartum	1.78 ± 0.14
Sady 1989 [93]	45	29 ± 4	26 weeks’ gestation	1.91 ± 0.32
			8 weeks postpartum	1.83 ± 0.31
Pregnant/non-pregnant control studies				
Bijl 2020 [85]	20	33.7 ± 4.3	11 weeks’ gestation	1.7 (1.5; 2.0) *^
	20	25.3 ± 1.9	Non-pregnant controls	2.2 (2.0; 2.5) *^
Heenan 2001 [81]	14	31 ± 1	35 weeks’ gestation	2.25 ± 0.10
	14	31 ± 2	Non-pregnant controls	2.28 ± 0.08
Sady 1988 [94]	40	29 ± 4	26 weeks’ gestation	1.89 ± 0.31
	10	30 ± 5	Non-pregnant controls	1.82 ± 1.21

*n*: number of female participants; VO_2max_: maximal oxygen consumption. L/min: litres per minute. Data are mean ± SD or mean (CI). * Data reported as L/kg/min. ^ Data reported as VO_2_ at 70% of maximum HR.

Additionally, the ventilatory threshold (VT; the point during exercise at which respiratory rate begins to increase more quickly than VO_2_) did not differ significantly between pregnant and non-pregnant women [81]. Boardman et al. discovered no significant difference between peak workload at 14 and 24 weeks’ gestation (165 ± 35.7 vs. 170 ± 36.9 Watts, *p* = 0.51) or resting mean arterial pressure [86]. The studies cited by Melzer et al., as well as some further studies, are tabulated (Table 4) [81,85,86,90,91,92,93,94]. Selected CPET parameters, as recorded by Jędrzejko et al. during late pregnancy, are shown in Appendix A Table A5 [82].

## 4. Discussion

### 4.1. Exercise Testing: The Current Literature

It is increasingly recognised that true functional capacity is better inferred by assessment of exercise tolerance than by resting cardiopulmonary function [10]. However, as demonstrated in the Results section, a comprehensive understanding of exercise tolerance for young women in functional exercise testing is still lacking.

In view of the data, constructing the most accurate reference ranges for these tests must encompass parameters including weight, height and BMI, sex, age, ethnicity and baseline reported physical activity. Combining outcome data from normal and above-weight BMI, or age spanning decades, will reduce data accuracy for any given population. Future study design should endeavour to separate female and male datasets within age groupings to maximise the applicability of their findings. Strict adherence to guidelines is critical to reduce error; 6MWT data demonstrate that added encouragement by the investigator improves distance walked, whilst a track layout with more turns decreases it [95]. It is recommended to perform two walks to allow for the averaging of data, as a learning effect exists (with the second walk improving on average by 12 m, an effect seen in 69% of subjects) [96].

Whilst each cohort of participants are intrinsically different anthropometrically, demographically and in fitness capacity—and thus, each reference range is distinct—there are singular studies which are representative of the expanse of published literature. Due to discrepancies in data reporting, pooling of data was not feasible, so representative studies were selected for use as a summary (Table 5). These selected studies included larger sample sizes, and outcome measures were similar to mean results across the spectrum of available data, making them exemplary illustrations.

However, there are limitations to the literature. As described above, biological sex and age have significant influences on the average distance walked in both the 6MWT and ISWT. A limitation of many studies is that there is no disaggregating by sex, rendering published reference values inaccurate; also, most studies do not present data in age grouping by decades, instead reporting data as all-inclusive age groups. Consequently, the most meticulous representation of data is through individual grouping of females and males by age group. This is done well in some studies, such as those referenced in Table 3 [63,64,65,66,67,68,69,70,71,72,73,74,75,76,77,78,79]. However, these did not divide women into active and sedentary categories (with Neder et al. using exclusively sedentary participants), which likely contributes to the increased standard deviations reported in these studies [67]. Importantly, some studies do not index VO_2max_ to body weight, which means that the combining of study data is not possible. A further modification to data reporting which would improve the interpretability of results is the use of consistent units of measurement that index for weight. Another limitation of the studies is the use of different summary values; currently, approximately half of studies use the mean and standard deviation, and half employ the median and interquartile range. This likely reflects the distribution of the data and whether or not it is skewed; a result of this is that the data are not readily able to be pooled.

### 4.2. The Safety of Exercise during Pregnancy

In the past, exercise was considered a theoretical threat to a developing fetus due to increased oxygen demand and substrate usage and increased heat and by-product production. It was thought that these environmental changes may cause teratogenesis in early pregnancy or lead to miscarriage, premature labour, or fetal distress due to hypoxia. However, evidence shows that the fetus can safely withstand exercise, and it is also important for pregnant women to exercise to maintain maternal health. Despite decisive recommendations from global obstetric authorities, only approximately one third of Australian pregnant women and one quarter of American pregnant women meet recommended activity levels [97,98]. Mother–child safety concerns are still the foremost perceived barrier to performing LTPA for pregnant women [99].

Unborn babies have been shown to cope well with maternal exercise. The monitoring of fetal heart rate (FHR) during maximal exercise in one study demonstrated mean FHR of 148 ± 7 bpm, consistent with pre-exercise FHR values (147 ± 6 bpm) and post-exercise rest values (143 ± 14 bpm) [100]. However, an incidence of bradycardia of 16.2% has been reported after maximal exercise, suggesting potential fetal hypoxia [100]. Thus, even though MacPhail et al. found that maximal maternal exertion in late gestation, such as cycle ergometer testing during CPET, is safe for brief periods of time, a recommendation for submaximal exertion during LTPA in pregnancy is preferable [101].

Notably, antenatal exercise has been shown to increase rates of vaginal delivery (RR 1.11, 95% CI (1.04–1.18); *p* = 0.051), lead to a shorter duration of active labour (mean difference 3.1 h, 95% CI (0.31–5.9); *p* = 0.029) and reduce the risk of a prolonged first stage of labour (9.8% in active vs. 19.4% in less active women; *p* < 0.01; adjusted RR 0.55; 95% CI (0.34–0.83)) [102,103,104]. Additionally, insufficient physical activity can increase the chance of Caesarean delivery for “medical reasons” (defined as fetal distress, cephalopelvic disproportion, non-cephalic fetal presentation, placenta praevia and more; adjusted OR 1.13, 95% CI (1.04–1.23)) but not for “non-medical reasons” (defined as social, familial or personal factors) [105].

Considering the significant morbidity, and sometimes mortality, associated with operative delivery or Caesarean section, encouraging LTPA during pregnancy can make a significant difference to maternal delivery outcomes. Furthermore, whilst there is currently no clear data about the influence of antenatal LTPA on recovery from Caesarean section, surgical “prehabilitation” has demonstrated patient benefits, such as improved postoperative pain and better physical function, and benefits to healthcare systems, such as decreased postoperative length of stay (Hedges’ g = −0.39, *p* = 0.033) [106]. Overall, the simple intervention of encouraging LTPA in pregnant women can, on an individual to global level, significantly improve healthcare outcomes.

## 5. Conclusions

There is a significant lack of data representing the responses of young pregnant and non-pregnant females to exercise testing in the domains of the Six Minute Walk Test, the Incremental Shuttle Walk Test and Cardiopulmonary Exercise Testing. This is a serious omission in the scientific literature, as exercise testing cannot be used appropriately in this population without accurate reference ranges. Clarifying these would offer bountiful utility in clinical practice, as exercise testing is a well-validated indicator of general health and disease progression. Exercise testing could be easily and cost-effectively harnessed as another tool in the clinician’s arsenal for patient assessment, such as in the formulation of a personalised antenatal exercise prescription, in order to encourage pregnant women to reap the health benefits of physical activity. With clear links between physical activity and preeclampsia, and the known benefits of exercise in the treatment of general hypertension, cardiac disease and operative outcomes, the understanding of exercise tolerance provided by this review allows clinicians to measurably assess progress with antenatal and postpartum exercise prescriptions. Importantly, the impact of antenatal exercise on perinatal outcomes, as well as long-term health for mother and child, is an area for further investigation, and this can be aided by the use of functional exercise testing techniques.

## Figures and Tables

**Table 5:** Summary of reference ranges of 6MWT, ISWT and CPET for non-pregnant and pregnant women.

Exercise Test	Outcome Measure
6MWT	Distance walked (m)
Non-pregnant (Kim 2014) [21]	581 ± 47.8
Early pregnancy (Dennis 2018) [37]	548 ± 80.9
Late pregnancy (Dennis 2018) [38]	488 ± 94.9
ISWT	Distance walked (m)
Non-pregnant (Agarwal 2016) [48]	709 ± 51.3
Pregnant	No available data
CPET	VO_2max_ (L/min)
Non-pregnant (Heenan 2001) [81]	2.28 ± 0.08
Pregnant (Heenan 2001) [81]	2.25 ± 0.10

6MWT: Six Minute Walk Test; ISWT: Incremental Shuttle Walk Test; CPET: Cardiopulmonary Exercise Testing; VO_2max_: maximal oxygen consumption. m: metres; L/min: litres per minute. Data are mean ± SD.

## Data Availability

No systematic review protocol was prepared for this investigation. No new data were created to make available for review.

## Appendix A

**Table A1:** Reference range studies for the Six Minute Walk Test in healthy non-pregnant adults.

Study	Female Participants (*n*)	Age (Years)	6MWD (m)
Halliday 2020 [26]	190	32.5 (27, 39) *	636 ± 88 *
Johnson 2020 [29]	69	23.0 ± 2.0	403.4 ± 91.6
Marques 2020 [25]	55	18–49	659 ± 81 ^
Oliveira 2019 [27]	89	39.8 ± 13.1	604.6 ± 66.8
Zou 2017 (ages 18–30) [35]	176	23.8 ± 3.77	607.4 ± 51.0
Zou 2017 (ages 18–59) [36]	319	40.0 ± 12.0	578 ± 49.9
Shrestha 2015 [30]	76	28.9 ± 13.2 *	445.1 ± 78.3
Kim 2014 [21]	164	37.7 ± 11.4	580.9 ± 47.8
Mosharraf-Hossain 2013 [24]	53	37.9 ± 8.5 *	466.7 ± 69.4 *
Rao 2013 [31]	85	38.7 ± 13.8	389.3 ± 74.3
Alameri 2009 [34]	111	30 ± 8	386 ± 45
Iwama 2009 [33]	73	35 (24, 52)	551 ± 71
Chetta 2006 [22]	54	33 ± 9	593 ± 57
Gibbons 2001 [23]	15	20–40	699 ± 37

*n*: number of female participants; 6MWD: Six Minute Walk Distance; m: metres. Data are mean ± SD, median (IQR), or lowest to highest value x-y. * Combined male and female data according to age group. ^ Combined data from age categories.

**Table A2:** Reference range studies for the Incremental Shuttle Walk Test in healthy non-pregnant adults.

Study	Female Participants (*n*)	Age (Years)	ISWD (m)
Marques 2020 [25]	52	18–49	1132 ± 334 ^
Lima 2019 [53]	54	26.4 ± 5.6	821.1 ± 118.9
Itaki 2018 [50]	120	28 (25, 33) *	750 (575, 915) *
Agarwal 2016 [48]	289	17–40	709.2 ± 51.3
Gonçalves 2015 [55]	29 32	22 (21, 24) * 35 (31, 37) *	1110 (1000, 1290) * 1010 (920, 1203) *
Harrison 2013 [51]	25	44.8 ± 2.98	824 ± 163
Seixas 2013 [54]	28	35.1 ± 12.53 *	958.3 ± 146.3 *
Probst 2012 [52]	140	51 (33, 66)	720 (480, 910)

*n*: number of participants; ISWD: Incremental Shuttle Walk Distance; m: metres. Data are mean ± SD, or median (IQR) or lowest to highest value x-y. ^: Combined data from age categories. *: Combined male and female data according to age group.

**Table A3:** Incremental Shuttle Walk Distance in women with pulmonary hypertension, before and during pregnancy.

Patient	1	2	3	4	5	6	7	8	8 *	9
Presentation ISWD (m)	450	330	140	400	130	370	0	0	260	200
Repeat ISWD (m)	450	440	130	420	90	-	470	350	-	340

ISWD: Incremental Shuttle Walk Distance; m: metres. *n* = 9 (with 10 pregnancies. *: Same participant upon second pregnancy). Data adapted from Kiely (2010) [57].

**Table A4:** Heart rate and blood pressure variables in non-pregnant females aged 20–29 years using cycle ergometry.

Time of Measurement	Heart Rate (bpm)	Systolic Blood Pressure (mmHg)
At rest	75.5 ± 9.8	112.1 ± 11.6
At anaerobic threshold	120.3 ± 12.9	131.6 ± 13.7
At peak exercise	171.3 ± 13.1	162.3 ± 14.1

Number of participants ≥ 44 for each parameter measured. bpm: beats per minute; mmHg: millimetres of Mercury. Data are mean ± SD. Data adapted from Itoh (2013) [80].

**Table A5:** Cardiopulmonary Exercise Testing-measured ergospirometric values in healthy late-pregnant women using cycle ergometry.

Cohort characteristics		
*n*	60	
Age (years)	24.4 ± 3.9	
Gestation (weeks)	37^+0^ to 41^+6^	
Ergospirometric parameters	Rest	Submaximal Exercise (75W)
HR (bpm)	90.1 ± 9.5	142.6 ± 10.8
VE (L/min)	6.7 ± 1.6	34.1 ± 4.6
RER	0.9 ± 0.1	1.2 ± 0.2
TV (L)	0.4 ± 0.1	1.2 ± 0.2
VO_2_ (L/min)	0.20 ± 0.07	0.88 ± 0.14
VCO_2_ (L/min)	0.18 ± 0.05	0.99 ± 0.13
EE (kJ/min)	4.3 ± 1.4	20.1 ± 3.7
MET	0.8 ± 0.3	3.4 ± 0.8
VO_2max_ (L/min)(extrapolated from submaximal values)	-	2.19 ± 0.33 (via ergospirometry)
	-	2.04 ± 0.25 (via pulsometry)

*n*: number of participants. Data are mean ± SD. W: Watt; HR: heart rate; bpm: beats per minute; VE: minute ventilation; L/min: litres per minute; RER: respiratory exchange ratio; TV: tidal volume; L: litres; VO_2_: oxygen uptake; VCO_2_: carbon dioxide output; EE: energy expenditure; kJ/min: kilojoules per minute; MET: metabolic equivalent; VO_2max_: maximal oxygen consumption. Data adapted from Jędrzejko (2016) [82].
